# The specificity of the Threat/Control-Override concept in schizophrenia – new insights from a retrospective cross-sectional study of forensic homicide offenders

**DOI:** 10.3389/fpsyt.2025.1658271

**Published:** 2025-10-08

**Authors:** Hannelore Findeis, Maria Strauß, Hans-Ludwig Kröber

**Affiliations:** ^1^ Klinik für Psychiatrie und Psychotherapie, Universitätsklinikum Leipzig, Leipzig, Germany; ^2^ Institut für forensische Psychiatrie, Charité Berlin, Berlin, Germany

**Keywords:** TCO, violence, schizophrenia, psychopathology, forensic psychiatry

## Abstract

**Introduction:**

There is evidence that there is a small group of people with schizophrenia spectrum disorders who are more likely to commit homicide than those in the general population. The aim of this study is to re-examine the much-discussed psychopathological concept of Threat/Control-Override with particular regard to its specificity for schizophrenia spectrum disorders, which has not been investigated to date.

**Methods:**

A file-based, retrospective, cross-sectional study was conducted to obtain a complete overview of all forensic homicide offenders detained in the Berlin Forensic Hospital as of 31 December 2014.

**Results:**

Of a total of 614 forensic patients, 110 committed homicide (17.9%). There are three main diagnostic groups in the forensic hospital who committed homicide: schizophrenia spectrum disorders (*n*=78), substance use disorders (*n*=11), and personality disorders (*n*=21). All patients were characterised by being male, unemployed and single. Both the total TCO complex (*p*=.001) and the Threat (*p*=.001) and Control-Override (*p*=.001) symptoms were statistically significantly more frequent in patients with schizophrenia spectrum disorders in the group comparison.

**Discussion:**

For the first time, the TCO complex is examined in a cross-diagnostic comparison, and the specificity of TCO for patients with schizophrenia spectrum disorders with the most serious violent offences can be demonstrated. In order to avoid false positives and to be able to identify clear psychopathological risk symptoms, future studies should include larger samples and, most importantly, non-offending controls.

## 1 Introduction

The worldwide prevalence of schizophrenic psychosis is 0.5 – 1%. In Germany, 0.5% of these patients are admitted to a forensic psychiatric hospital. This is therefore a comparatively small group. Patients with schizophrenia spectrum disorders are more likely to become victims of violence themselves (1). Nevertheless, the moderately significant association between schizophrenia and violence has been recognised since the seminal work of Häfner and Böker (2) and has been replicated many times since (3–7). In particular, there is evidence of an association between schizophrenia and the most serious violent crimes (6, 8) and homicide (6, 9–13).

Stompe (14) was able to show that 80% of patients with schizophrenia spectrum disorders who committed serious violent crimes had a delusional motive for committing the crime. These results are consistent with the frequently replicated findings that schizophrenic homicidal offenders have a particularly high psychopathological symptom burden at the time of the index offence (7, 11, 15–18). General crime factors played a greater role in minor offences than in serious violent crimes. Stompe and Schanda found that 65.9% of schizophrenic patients with minor offences had already exhibited criminal behaviour before the age of 14 (19).

In the 1990s, Link and Stueve (20) formulated a psychopathological symptom complex Threat/Control-Override (TCO), the presence of which represents a particular risk constellation for an imminent violent offence in patients with schizophrenia spectrum disorders. The authors defined Threat as a fear, for example through radiation or body hallucinations. Control-Override was defined as being controlled by external forces, such as thought withdrawal or insertion and being at the mercy of others.

The association between TCO and an increased potential for violent behaviour in patients with schizophrenia spectrum disorders was initially replicated in numerous studies (21–27). However, TCO has also been viewed critically: Mullen (28) criticised the fact that studies validating TCO have produced many false positives (TCO present in non-offenders), which the authors did not discuss sufficiently. In their large-scale *MacArthur Violence Risk Assessment Study*, Appelbaum et al. (29) found that the apparently significant association of TCO with an increased potential for violence in patients with schizophrenia spectrum disorders was no longer statistically significant when the covariates “anger” and “impulsivity” were included. In a study of the same dataset, Teasdale et al. (30) found that the presence of TCO in women was associated with significantly fewer violent offences. The authors posited that the negative findings reported by Appelbaum et al. (29) were attributable to the levelling effect of women (30).

Nederlof et al. (24) confirmed the TCO concept in their multi-centre cross-sectional study. However, the authors also found that a patient’s baseline disposition for the factors “fear” and “anger” was significantly associated with violent behaviour. In their meta-analysis and systematic review, Witt et al. (7) found no statistically significant association between TCO symptoms and violent behaviour, although it should be noted that the authors included aggressive and hostile verbal behaviour in violent behaviour, in contrast to the original TCO definition by Link and Stueve (20). In a recent discussion, Findeis et al. (31) explored two prevalent definitions of TCO within the German-language sphere (Stompe et al. (11) and Kröber (32)). They concluded that neither of them fits perfectly and suggested that a combination with proportions from both definitions could be a contribution to a future definition of TCO (31).

The retrospective comparative studies by Stompe et al. (11, 15 with delinquent and non-delinquent subjects with schizophrenia spectrum disorders initially showed that there was no statistically significant difference in the prevalence of TCO between the two groups. However, when an additional distinction was made between subjects who had committed serious violent offences and those who had committed minor violent offences, a statistically significant increase in TCO symptoms was observed in the former group (11, 15).

Despite the extensive analysis and discussion of the TCO concept in the literature, no study has yet demonstrated TCO as a specific schizophrenic psychopathology. To date, no studies have identified the specificity of TCO for schizophrenia spectrum disorders. A comparative study of patients from different diagnostic groups with serious violent offences has not yet been published.

The present study aims to test the specificity of the TCO complex for schizophrenia spectrum disorders by comparing patients from different diagnostic groups who have committed serious violent crimes. On the basis of the extant data, the following hypothesis may be postulated: There is a statistically significant accumulation of TCO in homicide offenders with schizophrenia spectrum disorders in comparison to those without such disorders.

## 2 Materials and methods

### 2.1 Study design

This study is a sub-analysis of a retrospective, cross-sectional, file-based study (33). Patient interviews were not conducted. All forensic homicide offenders admitted to the Berlin Forensic Hospital were analysed for sociodemographic characteristics and TCO symptoms and compared according to the three predominant diagnostic groups (Three subsamples). The diagnostic categorisation of the clinical pictures is based on the ICD - 10 (10. International Statistical Classification of Diseases and Related Health Problems). For technical and ethical reasons, it is not possible to conduct an experimental or quasi-experimental study in this context. The study is therefore based on a non-experimental ex-post facto design. The sample can be considered as a total coverage of the relevant population.

### 2.2 Ethics and data privacy

After approval by the Senate Department of Justice of the State of Berlin, the personal files were reversibly recorded and analysed in pseudonymised form in accordance with the data protection regulations for personal data pursuant to the currently valid General Data Protection Regulation ((EU) 2016/679; applicable from May 25, 2018). There was no personal exploration or written survey of the subjects. All data were analysed by the first author alone. To ensure good interrater reliability, the first author was trained by the last author before data collection began and was supervised throughout the data collection process.

It is not possible to individualise patients based on the analysed pseudonymised data.

The data were collected between January 2014 and November 2015. For this purpose, the medical records of subjects admitted to the Berlin Forensic Hospital were reviewed, using the following two documents as sources of information: the verdict on the index offence and the expert opinion on culpability.

### 2.3 Inclusion and exclusion criteria

The overall sample consists exclusively of male and female patients from the Berlin Forensic Hospital. All patients with attempted or completed homicide who were admitted to the Berlin Forensic Hospital on 31 December 2014 were included (*N* = 114).

Attempted murder is the attempted but unsuccessful killing of a person, committed with intent to kill and a characteristic of murder. Murder and attempted murder are regulated in the section 211 of the German Criminal Code (§ 211 StGB). Patients whose medical records did not contain the required information had to be excluded from the study (*n* = 3). In addition, patients whose principal diagnosis did not correspond to the three most common diagnostic groups of forensic homicide offenders were excluded. This applied to one subject with the principal diagnosis of ICD-10 F07.8 (other organic personality and behavioural disorder due to disease, damage or dysfunction of the brain; *n* = 1).

**Figure 1:**
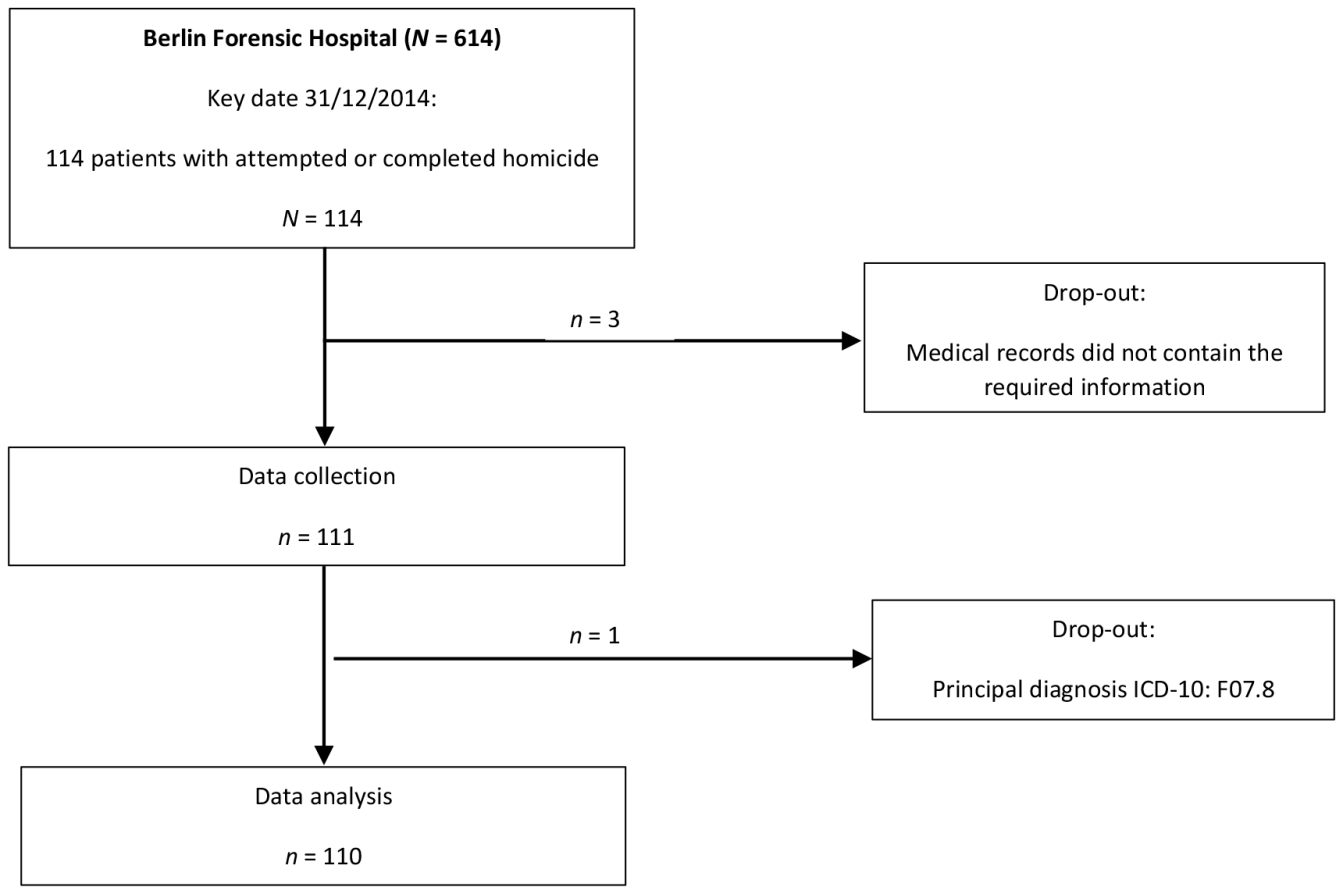
Flow diagram for selecting the patient records included in the survey. N/n – sample size; ICD-10 – 10. International Statistical Classification of Diseases and Related Health Problems.

In accordance with the inclusion and exclusion criteria, the sample consists of 110 patients with attempted or completed homicide offences from the Berlin Forensic Hospital (*n* = 110; **Figure 1** (33)).

### 2.4 Statistical analysis

The three subsamples were compared on 18 variables. All statistical analyses were performed using SPSS software (IBM SPSS Statistics, for Mac, version 29.0). The significance level (*α*) was set at .05 for all statistical tests.

The metric variable was tested for normal distribution using the Shapiro-Wilk test and visual inspection of the histograms for skewness and kurtosis. Homogeneity of variance was tested using the Levene test. As the assumption of normal distribution was violated, the non-parametric Kruskal-Wallis test was used.

Categorical variables were tested for statistically significant differences between the three diagnostic groups using Pearson’s *χ2* test. If the assumptions for Pearson’s *χ2* test were violated because the expected cell frequencies were too low, Fisher’s exact test was used. If the results were statistically significant, a pairwise comparison was then calculated as a *post-hoc* test using the *χ2* test or, if the assumptions were violated, Fisher’s exact test. For statistically significant results, the effect size was reported by calculating the Phi coefficient (Φ) or Cramer’s V (V). Values between 0.1 and less than 0.3 indicate a weak effect, values between 0.3 and less than 0.5 indicate a moderate effect and values greater than 0.5 indicate a strong effect. Due to the exploratory nature of the study, *p*-value adjustment for multiple testing was not performed.

### 2.5 Survey tools

All psychopathological symptoms were operationalised according to the AMDP system (34; Arbeitsgemeinschaft Für Methodik Und Dokumentation in Der Psychiatrie) (Arbeitsgemeinschaft für Methodik und Dokumentation in der Psychiatrie). The AMDP provides an international standard for the uniform recording of psychopathological findings, physical symptoms and medical history in patients with mental illness. It is the only standardised psychopathological diagnostic system listed in the German medical training regulations for psychiatry and psychotherapy.

The TCO definition used in this paper is that of Stompe et al. (11). This definition is the most up-to-date and is based on a study that differentiates between the severity of the offences. It is particularly suitable for the sample in this study. Stompe et al. (11, 15) retrospectively compared offenders with schizophrenia spectrum disorders with serious and minor offences and non-offenders. There was a statistically significant accumulation of TCO symptoms only in the group of serious violent offenders. Threat symptoms were defined as a particularly threatening form of persecutory delusion in which the patient is convinced that life and limb are acutely threatened. The prevalence of Threat symptoms was 70.7 % in serious offenders, 16.7 % in minor offenders and 46.1 % in non-offenders with schizophrenia spectrum disorders.

Control-Override symptoms were defined on the basis of Kurt Schneider’s first-order symptoms with thought withdrawal and/or thought insertion, as well as the delusional belief of being controlled by external forces (11).

**Table 1** (31, 33) summarises the definition of the TCO complex by Stompe et al. (11) and the AMDP operationalisations used in this study. Threat and Control-Override are considered to be met if all assigned variables apply.

**Table 1:** Operationalisation of TCO symptoms referring to Stompe et al. (11).

TCO symptoms	Definition referring to Stompe et al. (11)	Operationalisation
Threat	- Systematic delusion of persecution or poisoning concomitant with massive death threat by particular people or groups of people	1. Persecutory delusion or delusion of poisoning 2. Systematic delusion 3. Hostile and destructive delusion 4. Highly affective involvement in the delusion
Control-Override	- Thought withdrawal - Thought insertion - Delusional belief that external powers are in control of one´s own emotions, actions and desires	5. Thought withdrawal/insertion

TCO, Threat/Control-Override.

## 3 Results

### 3.1 Sociodemography

The results for the socio-demographic variables are presented in **Table 2**. The three diagnosis groups differed statistically significantly and with a large effect size from each other with regard to the applied articles of criminal responsibility (§§ 20 and 21 StGB; *p* = .001; V = .618) as well as with regard to the applied articles of detention in a forensic hospital (§§ 63 and 64 StGB; *p* = .001; V = .896). The *post-hoc* test showed that the subjects with schizophrenia spectrum disorders were statistically significantly more often sentenced according to § 20 StGB than the personality disordered (*p* = .001; Φ = .614) and addicted (*p* = .001; Φ = .494) subjects. It was also found that the addicted subjects were statistically significantly more likely to be sentenced under § 64 StGB than the personality disordered subjects (*p* = .001; Φ = .864) and subjects with schizophrenia spectrum disorders (*p* = .001; Φ = .893). There are strong effect sizes in each case, although the effect size of the statistically significant result for the subjects with schizophrenia spectrum disorders compared to the addicted subjects was only marginally strong (Φ = .494) for the article of criminal responsibility.

**Table 2:** Sociodemographic variables (n = 110).

Variable	Value	F1x *n =* 11	F2x *n =* 78	F6x *n =* 21	Statistics	*p;* Φ/V	*p* post-hoc-test; Φ/V
Age at index offence, *M (SD), spread, median*		34.5 (11.3), 34.0, 30.0	32.5 (9.6), 48.0, 29.0	29.8 (10.1), 35.0, 26.0	*z* = 2.3	.304	
Sex, *n (%)*	Male	9 (81.8)	69 (88.5)	20 (95.2)	*[Exact Fisher Test]* = 1.518	.479	
	Female	2 (18.2)	9 (11.5)	1 (4.8)			
Index offence, *n (%)*	Murder	0 (0.0)	9 (11.5)	10 (47.6)	*[Exact Fisher Test]* = 24.537	.001**; .334	F1x – F2x .433 F1x – F6x .001**; .700 F2x – F6x .001**; .439
	Attempted murder	3 (27.3)	10 (12.8)	3 (14.3)			
	Manslaughter	3 (27.3)	30 (38.5)	8 (38.1)			
	Attempted manslaughter	5 (45.5)	29 (37.2)	0 (0.0)			
Marital status, *n (%)*	Relationship	3 (27.3)	17 (21.8)	6 (28.6)	*[Exact Fisher Test]* = 0.754	.713	
	No relationship	8 (72.7)	61 (78.2)	15 (71.4)			
Living status, *n (%)*	Proprietary apartment	8 (72.7)	54 (69.2)	16 (76.2)	*[Exact Fisher Test]* = 2.758	.614	
	Home	0 (0.0)	11 (14.1)	3 (14.3)			
	Homeless	3 (27.3)	13 (16.7)	2 (9.5)			
Occupational status, *n (%)*	Unemployed	10 (90.9)	62 (79.5)	16 (76.2)	*[Exact Fisher Test]* = 2.596	.881	
	Retirement pension	0 (0.0)	7 (9.0)	1 (4.8)			
	Employed	1 (9.1)	5 (6.4)	2 (9.5)			
	Studies/training	0 (0.0)	4 (5.1)	2 (9.5)			
Financial status, *n (%)*	Proprietary income	0 (0.0)	8 (10.3)	5 (23.8)	*[Exact Fisher Test]* = 5.343	.213	
	Receipt of benefits or pensions	11 (100.0)	59 (75.6)	14 (66.7)			
	No income	0 (0.0)	11 (14.1)	2 (9.5)			
Nationality, *n (%)*	German	9 (81.8)	45 (57.7)	18 (85.7)	*[Exact Fisher Test]* = 6.720	.118	
	German with migration background	0 (0.0)	9 (11.5)	0 (0.0)			
	Not german	2 (18.2)	24 (30.8)	3 (14.3)			
Article detainment forensic hospital, *n (%)*	§ 63 StGB	2 (18.2)	78 (100.0)	21 (100.0)	*[Exact Fisher Test]* = 46.721	.001**; .896	F1x – õF2x .001**; .893 F1x – F6x .001**; .864 F2x – F6x *a*
	§ 64 StGB	9 (81.8)	0 (0.0)	0 (0.0)			
Article criminal responsibility, *n (%)*	§ 20 StGB	4 (36.4)	71 (91.0)	6 (28.6)	*[Exact Fisher Test]* = 39.709	.001**; .618	F1x – F2x .001**; .494 F1x – F6x .703 F2x – F6x .001**; .614
	§ 21 StGB	7 (63.6)	7 (9.0)	15 (71.4)			
Graduation status*, n (%)*	No graduation	6 (54.5)	20 (25.6)	8 (38.1)	*[Exact Fisher Test]* = 4.846	.280	
	Secondary school level	5 (45.5)	47 (60.3)	10 (47.6)			
	General qualification for university entrance	0 (0.0)	11 (14.1)	3 (14.3)			
Higher educational status, *n (%)*	No higher education	9 (81.8)	50 (64.1)	10 (47.6)	*[Exact Fisher Test]* = 8.703	.046*; .191	F1x – F2x .074 F1x – F6x .134 F2x – F6x .089
	Completed vocational education	1 (9.1)	27 (34.6)	9 (42.9)			
	Completed studies	1 (9.1)	1 (1.3)	2 (9.5)			

*n*, sample size; *M*, mean; *SD*, standard deviation; F1x, substance use disorders; F2x, schizophrenia spectrum disorders; F6x, personality disorders; StGB, Strafgesetzbuch (German Criminal Code); *a*, no statistics are calculated as the variable is a constant; *p*, significance value; Φ/V, effect size; * <.05; ** <.01.

There was a statistically significant difference with a moderate effect size between the three groups regarding the index offence (*p* = .001; V = .334). The *post-hoc* test showed that the patients with personality disorders committed homicide statistically significantly more often than the patients with schizophrenia spectrum disorders (*p* = .001; Φ = .439) with a moderate effect size and then the addicted patients (*p* = .001; Φ = .700) with a strong effect size.

There was also a statistically significant difference between the groups in terms of education at the time of the index offence (*p* = .046; V = .191), with a small effect size. However, pairwise comparisons showed no statistically significant difference between the groups.

### 3.2 Psychopathology

The results for the psychopathological variables at the time of the index offence are shown in **Table 3**. There was a statistically significant difference with a large effect size between the three groups in terms of highly affective involvement in the delusion (*p* = .001; V = .548). The *post-hoc* test showed that the patients with schizophrenia spectrum disorders were statistically significantly more likely to have highly affective involvement in their delusion during the commission of the index offence than the patients with substance use disorders (*p* = .002; Φ = .348) with a moderate effect size and the patients with personality disorders (*p* = .001; Φ = .530) with a strong effect size.

**Table 3:** Psychopathology at time of index offence (n = 110).

Variable	F1x *n =* 11	F2x *n =* 78	F6x *n =* 21	Statistics	*p;* Φ/V	*p post-hoc*-test; Φ/V
Highly affective involvement in the delusion, *n (%)*	2 (18.2)	54 (69.2)	1 (4.8)	*[Exact Fisher Test]* = 35.455	.001**; .548	F1x – F2x .002**; .348 F1x – F6x .266 F2x – F6x .001**; .530
Delusion of poisoning, *n (%)*	1 (9.1)	21 (26.9)	0 (0.0)	*[Exact Fisher Test] * = 9.178	.005**; .276	F1x – F2x .280 F1x – F6x .344 F2x – F6x .005**; .269
Systematic delusion, *n (%)*	1 (9.1)	57 (73.1)	1 (4.8)	*[Exact Fisher Test]* = 43.591	.001**; .609	F1x – F2x .001**; .442 F1x – F6x 1.00 F2x – F6x .001**; .567
Thought withdrawal/insertion, *n (%)*	1 (9.1)	44 (56.4)	0 (0.0)	*[Exact Fisher Test]* = 30.935	.001**; .495	F1x – F2x .004**; .311 F1x – F6x .111 F2x – F6x .001**; .464
Persecutory delusion*, n (%)*	1 (9.1)	40 (51.3)	1 (4.8)	*[Exact Fisher Test]* = 21.152	.001**; .422	F1x – F2x .010*; .279 F1 – F6x 1.00 F2x – F6x.001**; .386
Hostile and destructive delusion, *n (%)*	1 (9.1)	55 (70.5)	2 (9.5)	*[Exact Fisher Test]* = 35.550	.001**; .556	F1x – F2x .001**; .419 F1x – F6x 1.00 F2x – F6x .001**; .504

*n*, sample size; *M*, mean; *SD*, standard deviation; F1x, substance use disorders; F2x, schizophrenia spectrum disorders, F6x, personality disorders; *p*, significance value; Φ/V, effect size; * <.05; ** <.01. TCO symptoms (n = 110). *n* – sample size; TCO – Threat/Control-Override.

There was a statistically significant difference between the groups with regard to the delusion of poisoning (*p* = .005; V = .276). The *post-hoc* test showed that the patients with schizophrenia spectrum disorders were statistically significantly more likely to have had a delusion of poisoning during the commission of the index offence than the patients with personality disorders (*p* = .005; Φ = .269), who didn’t have a delusion of poisoning at all. Both effect sizes are small to moderate.

There was a statistically significant difference with a strong effect size between the three groups with regard to systematic delusion (*p* = .001; V = .609). The *post-hoc* test showed that the patients with schizophrenia spectrum disorders were statistically significantly more likely to have a systematic delusion during the commission of the index offence than the patients with substance use disorders with a moderate effect size (*p* = .001; Φ = .442) and the patients with personality disorders with a strong effect size (*p* = .001; Φ = .567).

A statistically significant difference was identified between the three groups with regard to thought withdrawal or insertion (*p* = .001; V = .495). The *post-hoc* test demonstrated, with a moderate effect size, that, in comparison to the addicted patients (*p* = .004; Φ = .311) and the personality-disordered patients (*p* = .001; Φ = .464), patients with schizophrenia spectrum disorders experienced thought withdrawal or insertion with a higher frequency during the commission of the index offence.

A statistically significant difference was observed between the groups with regard to persecutory delusions (*p* = .001; V = .422), exhibiting a moderate effect size. The *post-hoc* test demonstrated that patients diagnosed with a schizophrenia spectrum disorder were statistically significantly more likely to experience persecutory delusions in comparison to patients with substance use disorders (*p* = .010; Φ = .279) with an almost moderate effect size and personality disorders (*p* = .001; Φ = .386) with a moderate effect size.

A statistically significant difference was observed between the groups with regard to the presence of hostile and destructive delusions (*p* = .001; V = .556). The *post-hoc* test demonstrated with approximate moderate effect sizes that patients diagnosed with schizophrenia spectrum disorders were statistically significantly more likely to experience hostile and destructive delusions during the commission of the index offence in comparison to patients with a substance use disorder (*p* = .001; Φ = .419) and those with a personality disorder (*p* = .001; Φ = .504).

### 3.3 Threat/control-override

**Figure 2:**
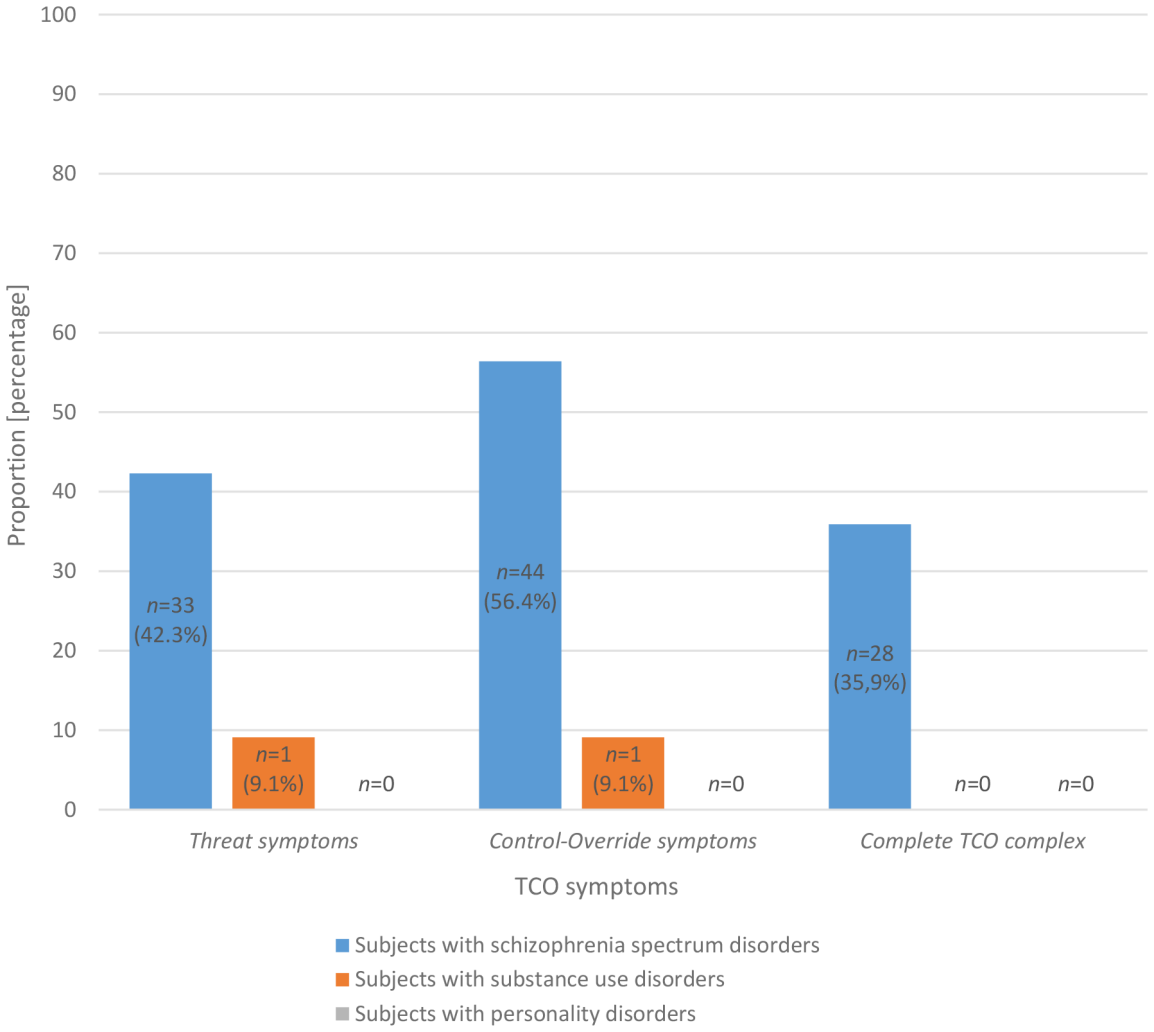
TCO symptoms (n = 110). n – sample size; TCO – Threat/Control-Override.

The results are presented in **Figure 2**. A statistically significant difference was identified between the three groups with regard to the presence of Threat symptoms (*p* = .001; V = .388). The *post-hoc* test revealed that patients diagnosed with schizophrenia were statistically significantly more likely to manifest Threat symptoms in comparison to patients with substance use disorders (*p* = .046; V = .225), exhibiting a weak effect size, and patients with personality disorders (*p* = .001; V = .367), demonstrating a moderate effect size.

A statistically significant difference was also identified among the three groups with respect to the presence of Control-Override symptoms (*p* = .001; V = .495). The *post-hoc* test revealed that patients diagnosed with schizophrenia were statistically significantly more likely to exhibit Control-Override symptoms in comparison to patients with substance use disorders (*p* = .004; V = .311) and personality disorders (*p* = .001; V = .464), with a moderate effect size. The complete TCO complex was observed in 35.9% of subjects diagnosed with a schizophrenia spectrum disorder and in none of the subjects diagnosed with a substance use disorder or a personality disorder. Accordingly, a statistically significant difference was observed between the three groups regarding the presence of the complete TCO complex (*p* = .001; V = .374). The *post-hoc* test revealed a statistically significant difference for patients with substance use disorders (*p* = .015; V = .254) with an almost moderate effect size and for patients with personality disorders (*p* = .001; V = .326) with a moderate effect size.

## 4 Discussion

The present study examines a sample of forensic homicide offenders, of whom two third were found to have schizophrenia spectrum disorders. This figure is slightly below the 70 – 80 % reported in the literature on the subject (6, 35, 36). At the time of the study, 21 % of all patients in the Berlin Forensic Hospital suffered from a substance use disorder, compared to around 18 % of patients detained under § 64 StGB, as reported in the literature. The slight discrepancy in these figures can be attributed to the use of the detainment article in the literature and the main psychiatric diagnoses in the present study. In cases, patients diagnosed with a substance use disorder are also detained under § 63 StGB. According to Müller et al. (36), at the time of the study, approximately one in ten patients admitted under § 63 StGB at the Berlin Forensic Hospital had a primary diagnosis of personality disorder. However, when only homicide offenders are considered, this ratio is significantly different: The proportion of patients diagnosed with a personality disorder who had committed homicide was almost one in five, and of all personality disorder patients at the Berlin Forensic Hospital, almost one in two had committed homicide. In comparison, about one in five patients with schizophrenia spectrum disorders and about one in ten addicted patients had committed a homicide.

Across all diagnoses, the majority of homicide offenders were male, unemployed and single. However, homicide offenders with personality disorders were more frequent to have a home and were the least frequent to be homeless at the time of the offence compared to the other two groups. This also applied to their financial situation; twice as many personality-disordered as subjects with schizophrenia spectrum disorders were employed and had their own income, and over half of the subjects had completed vocational education or a higher degree. These findings suggest that subjects with personality disorders were more socially integrated at the time of the index offence than those with substance use disorders or schizophrenia. Furthermore, more than half of those with personality disorders had committed attempted or completed murder; there was a statistically significant difference between this group and the other two diagnostic groups.

In contrast, patients with schizophrenia spectrum disorders were found to be more frequently incapable of guilt (conviction according to § 20 StGB) when committing their index offence than those with substance abuse or personality disorders. This result anticipates an important finding about the subsample of subjects with schizophrenia spectrum disorders: the particularly marked psychopathology of offenders with schizophrenia spectrum disorders leads to almost exclusive inculpability.

The most prevalent psychopathological symptoms were identified as a highly affective involvement in the delusion and systematised and hostile-destructive delusions. The results align with the frequently replicated findings that individuals diagnosed with schizophrenia spectrum disorders who have committed homicide offences exhibit a notably elevated psychopathological symptom burden at the time of the offence (7, 11, 15–18). Stompe et al. (11, 15) described the same most common specific symptoms in patients with the most serious violent offences: systematised delusions and a highly affective involvement in the delusion.

Although none of the psychopathological symptoms occurred exclusively in patients with schizophrenia spectrum disorders, there was a statistically significant difference in the occurrence of all symptoms compared to the other two diagnosis groups. Notably, the occurrence of delusions of poisoning exhibited no statistical significance between subjects with substance use disorder and those diagnosed with schizophrenia, likely attributable to the limited subsample sizes. In addition, there is a patient with a primary diagnosis of a substance use disorder and a corresponding conviction under § 64 StGB, whose predominant psychopathology at the time of the offence and the longitudinal course of the illness suggest a schizophrenia spectrum disorder as a differential diagnosis. This illustrates a frequently challenging dilemma: the diagnosis of a substance-induced psychotic disorder as distinct from a schizophrenia spectrum disorder. The differential diagnosis of paranoid schizophrenia has previously been discussed in the subject’s expert opinion on culpability, and this diagnosis is supported by the typical psychopathology and the age of onset. In addition, there were hardly any reports of long-term substance use in the medical records. Rather, the expert opinion indicates that the patient mainly consumed substances (questionably in the sense of self-medication) in moments of great psychotically motivated anxiety and a highly affective involvement in the delusion. However, given that the patient had never been abstinent from addictive substances for a minimum of six months since his youth, the possibility of a substance-induced psychotic disorder (ICD-10 F15.5, drug-induced psychosis) could not be formally excluded, which is why this patient was detained under § 64 StGB.

In terms of TCO, the full TCO complex (35.9 %) was less common in the present study than in Stompe et al. (11), where 52.0 % of subjects with the most serious offences exhibited the complete TCO complex at the time of the offence. As a result, the symptoms of Threat (42.3 % vs. 70.7 %) and Control-Override (56.4 % vs. 64.0 %) are also less frequent in the present study. This difference may be explained by the strict definition of TCO symptoms in this study, as both Threat and Control-Override were only considered to be present if all operationalised symptoms were present. It remains open whether Stompe et al. (11) considered Threat/Control-Overrride to be fulfilled if at least one of the psychopathological symptoms mentioned applied. And considering the higher prevalence rates reported by Stompe et al. (11), it stands to reason that they applied a less strict definition of TCO. It should be noted that the available literature does not yet clarify how many symptoms of Threat and Control-Override must be present for TCO to be met. The present approach therefore represents a possible operationalisation. A comprehensive discussion of common TCO definitions can be found in Findeis et al. (31).

The findings of the present study demonstrate a previously unpublished statistically significant accumulation of both Threat and Control-Override symptoms as well as the entire TCO complex in the homicide offenders with schizophrenia spectrum disorders when compared to other diagnostic groups. This is the first time that the specificity of the psychopathological symptom complex TCO has been systematically investigated by comparing different diagnostic groups. The symptoms documented in TCO do not indicate a general elevated risk of violence in individuals with mental illness; rather, they are indicative of a distinct and specific schizophrenic psychopathology.

The data presented in this study was obtained exclusively from the verdict on the index offence and the expert opinion on culpability of the respective subjects. It is not possible to ascertain whether pertinent information regarding the subjects is absent due to incomplete documentation, and as the sources of information have different authors, it is not possible to rule out the possibility of bias effects in the assessment of the variables.

The absence of personal interviews with the subjects may represent a limitation. Conversely, the exclusive analysis of the case files also represents a methodological strength of the study: As only the comprehensive development of the subjects up to the index offence is relevant for the questions and the expert reports were in most cases drawn up very soon after the offence, the psychopathology of the subjects could be adequately depicted. Personal interviews would have meant a strong distortion of the patient reports due to the index offences having occurred many years previously.

To ensure good interrater reliability, the first author was trained by the last author before data collection began and was supervised throughout the entire data collection process. However, it should be noted that no interrater reliability was calculated, which may be a limitation of the study.

The study represents an overall survey, which is another strength. The comparative study of homicide offenders against the background of their various principal diagnoses is a previously unpublished study design.

Due to the insufficient number of female subjects (*n*=12), the data of the male and female subjects were recorded and statistically analysed together in this study.

The total sample (*n* = 110) is comprised of two relatively small subsamples (*n*_F1x_ = 11; *n*_F6x_ = 21) and a larger subsample (*n*_F2x_ = 78), which poses problems in terms of test strength. The presence of actual differences between the groups is more challenging to ascertain, as the standard errors tend to be larger in cases of small sample sizes. Additionally, due to the insufficient size and marked variation of the subsamples, it was not feasible to compute a regression analysis with covariate adjustment for any predictive effects of individual variables. This limits the ability to control for confounding variables such as age, gender, or socio-economic status. Consequently, the interpretation and analysis of the present results were undertaken with particular caution.

It is recommended that future studies on these issues be conducted with larger samples in order to reduce bias and allow for predictive effects with appropriate covariate adjustments to control for confounding variables such as age, gender, or socio-economic status. To strengthen the validity of the TCO concept, future studies should include samples with delinquent and non-delinquent subjects with schizophrenia spectrum disorders.

In addition, it has been demonstrated that the relative risk of violence for women with schizophrenia spectrum disorders is significantly higher than for men (5, 37). This gender effect has also been observed in the two other diagnostic groups (38). These data suggest that gender-specific analyses should be carried out in future studies with larger samples.

## Data Availability

The raw data supporting the conclusions of this article will be made available by the authors, without undue reservation.

## References

[1] De Vries B Van Busschbach JT van der Stouwe ECD Aleman A Van Dijk JJM Lysaker PH. Prevalence rate and risk factors of victimization in adult patients with a psychotic disorder: A systematic review and meta-analysis. Schizophr Bull. (2019) 45:114–26. doi: 10.1093/SCHBUL/SBY020, PMID: 29547958 PMC6293237

[2] Häfner H Böker W. Gewalttaten Geistesgestörter. Berlin: Springer (1973). doi: 10.1007/978-3-642-86859-7

[3] Curic S. Psychiatrischer Beitrag: Korrelate zukünftiger Gewalt bei Personen, die wegen einer Schizophrenie behandelt werden. Forensische Psychiatrie Psychologie Kriminologie. (2019) 13:395–7. doi: 10.1007/S11757-019-00562-3

[4] Eronen M Hakola P Tiihonen J. Factors associated with homicide recidivism in a 13-year sample of homicide offenders in Finland. Psychiatr Serv. (1996) 47:403–6. doi: 10.1176/PS.47.4.403, PMID: 8689372

[5] Fazel S Gulati G Linsell L Geddes JR Grann M. Schizophrenia and violence: systematic review and meta-analysis. PLoS Med. (2009) 6. doi: 10.1371/JOURNAL.PMED.1000120, PMID: 19668362 PMC2718581

[6] Schanda H Knecht G Schreinze D Stompe T Ortwein-Swoboda G Waldhoer T. Homicide and major mental disorders: a 25-year study. Acta Psychiatrica Scandinavica. (2004) 110:98–107. doi: 10.1111/J.1600-0047.2004.00305.X, PMID: 15233710

[7] Witt K van Dorn R Fazel S. Risk factors for violence in psychosis: systematic review and meta-regression analysis of 110 studies. PLoS One. (2013) 8. doi: 10.1371/JOURNAL.PONE.0055942, PMID: 23418482 PMC3572179

[8] Taylor PJ. Motives for offending among violent and psychotic men. Br J Psychiatry : J Ment Sci. (1985) 147:491–8. doi: 10.1192/BJP.147.5.491, PMID: 4075044

[9] Erb M Hodgins S Freese R Müller-Isberner R Jöckel D. Homicide and schizophrenia: maybe treatment does have a preventive effect. Criminal Behav Ment Health : CBMH. (2001) 11:6–26. doi: 10.1002/CBM.366, PMID: 12048536

[10] Eronen M Hakola P Tiihonen J. Mental disorders and homicidal behavior in Finland. Arch Gen Psychiatry. (1996) 53:497–501. doi: 10.1001/ARCHPSYC.1996.01830060039005, PMID: 8639032

[11] Stompe T Ritter K Schanda H. Prädiktoren für Gewaltdelikte bei Schizophrenie. In: Stompe T Schanda H, editors. Schizophrenie und Gewalt. Medizinisch wissenschaftliche Verlagsgesellschaft, Berlin (2018). p. 91–148.

[12] Wallace C Mullen P Burgess P Palmer S Ruschena D Browne C. Serious criminal offending and mental disorder. Case linkage study. Br J Psychiatry : J Ment Sci. (1998) 172:477–84. doi: 10.1192/BJP.172.6.477, PMID: 9828986

[13] Whiting D Gulati G Geddes JR Fazel S. Association of schizophrenia spectrum disorders and violence perpetration in adults and adolescents from 15 countries: A systematic review and meta-analysis. JAMA Psychiatry. (2022) 79:120–32. doi: 10.1001/JAMAPSYCHIATRY.2021.3721, PMID: 34935869 PMC8696689

[14] Stompe T. Die Bedeutung des Wahns für die Risikoeinschätzung delinquenten Verhaltens schizophrener Patienten//Schizophrenia and Violence – The Impact of Delusions on Risk Assessment. J Für Neurologie Neurochirurgie Und Psychiatr. (2018) 19:104–10. doi: 10.1093/OXFORDJOURNALS.SCHBUL.A007066, PMID: 15176760

[15] Stompe T Ortwein-Swoboda G Schanda H. Schizophrenia, delusional symptoms, and violence: the threat/control override concept reexamined. Schizophr Bull. (2004) 30:31–44. doi: 10.1093/OXFORDJOURNALS.SCHBUL.A007066, PMID: 15176760

[16] Buchanan A Reed A Wessely S Garety P Taylor P Grubin D. Acting on delusions. II: the phenomenological correlates of acting on delusions. Br J Psychiatry. (1993) 163:77–81. doi: 10.1192/BJP.163.1.77, PMID: 8353704

[17] Prüter C. Zusammenhang zwischen Wahn und Gewalt. Gibt es stereotype Delikte bei Wahnkranken? In: Lammel M Sutarski S Lau S Bauer M, editors. Wahn und Schizophrenie. Psychopathologie und forensische Relevanz. Berlin: Medizinisch wissenschaftliche Verlagsgesellschaft (2011). p. 101–10.

[18] Swanson JW Swartz MS Van Dorn RA Elbogen EB Wagner HR Rosenheck RA. A national study of violent behavior in persons with schizophrenia. Arch Gen Psychiatry. (2006) 63:490–9. doi: 10.1001/ARCHPSYC.63.5.490, PMID: 16651506

[19] Stompe T Schanda H. Tatmerkmale der Tötungsdelikte von Patienten mit Schizophrenie. In: Stompe T Schanda (Hrsg.) H, editors. Schizophrenie und Gewalt. Medizinisch wissenschaftliche Verlagsgesellschaft, Berlin (2018). p. 155–73.

[20] Link BG Stueve A. Psychotic symptoms and the violent/illegal behavior of mental patients compared to community controls. In: Monahan J Steadman HJ, editors. Violence and mental disorder: Developments in risk assessment. The University of Chicago Press, chicago (1994). p. 137–59.

[21] Joyal CC Putkonen A Mancini-Marïe A Hodgins S Kononen M Boulay L. Violent persons with schizophrenia and comorbid disorders: a functional magnetic resonance imaging study. Schizophr Res. (2007) 91:97–102. doi: 10.1016/J.SCHRES.2006.12.014, PMID: 17291724

[22] Link BG Stueve A Phelan J. Psychotic symptoms and violent behaviors: probing the components of ‘threat/control-override’ symptoms. Soc Psychiatry Psychiatr Epidemiol. (1998) 33 Suppl 1. doi: 10.1007/S001270050210, PMID: 9857780

[23] Link BG Monahan J Stueve A Cullen FT. Real in their consequences: A sociological approach to understanding the association between psychotic symptoms and violence. Am Sociological Rev. (1999) 64:316–32. doi: 10.2307/2657535

[24] Nederlof AF Muris P Hovens JE. Threat/control-override symptoms and emotional reactions to positive symptoms as correlates of aggressive behavior in psychotic patients. J Nervous Ment Dis. (2011) 199:342–7. doi: 10.1097/NMD.0B013E3182175167, PMID: 21543954

[25] Nordström A Dahlgren L Kullgren G. Victim relations and factors triggering homicides committed by offenders with schizophrenia. J Forensic Psychiatry Psychol. (2006) 17:192–203. doi: 10.1080/14789940600631522

[26] Swanson J Estroff S Swartz M Borum R Lachicotte W Zimmer C. Violence and severe mental disorder in clinical and community populations: the effects of psychotic symptoms, comorbidity, and lack of treatment. Psychiatry. (1997) 60:1–22. doi: 10.1080/00332747.1997.11024781, PMID: 9130311

[27] Swanson JW Borum R Swartz MS Monahan J. Psychotic symptoms and disorders and the risk of violent behaviour in the community. Criminal Behav Ment Health. (1996) 6:309–29. doi: 10.1002/CBM.118

[28] Mullen PE. A reassessment of the link between mental disorder and violent behaviour, and its implications for clinical practice. Aust New Z J Psychiatry. (1997) 31:3–11. doi: 10.3109/00048679709073793, PMID: 9088480

[29] Appelbaum PS Robbins PC Monahan J. Violence and delusions: data from the MacArthur Violence Risk Assessment Study. Am J Psychiatry. (2000) 157:566–72. doi: 10.1176/APPI.AJP.157.4.566, PMID: 10739415

[30] Teasdale B Silver E Monahan J. Gender, threat/control-override delusions and violence. Law Hum Behav. (2006) 30:649–58. doi: 10.1007/S10979-006-9044-X, PMID: 16967327

[31] Findeis H Strauß M Kröber HL. The TCO concept in German forensic homicide offenders with schizophrenia spectrum disorders – new findings from a file-based, retrospective cross-sectional study. Front Psychiatry. (2024) 15:1404263/FULL. doi: 10.3389/FPSYT.2024.1404263/FULL, PMID: 38919633 PMC11196989

[32] Kröber HL. Kann man die akute Gefährlichkeit schizophren Erkrankter erkennen? Forensische Psychiatrie, Psychologie, Kriminologie, (2018) 2(2):128–36. doi: 10.1007/S11757-008-0073-9

[33] Findeis H. Patienten mit Tötungsdelikten im Berliner Krankenhaus des Maßregelvollzugs – Untersuchung des Zusammenhangs zwischen Tat und psychischer Störung oder Krankheit, Charité Berlin. (2024). doi: 10.17169/REFUBIUM-44529.

[34] AMDP. (2018) Arbeitsgemeinschaft für Methodik und Dokumentation in der Psychiatrie. Göttingen: AMDP. Available online at: https://www.amdp.de/ (Accessed April 25, 2023).

[35] Haller R Kemmler G Kocsis E Maetzler W Prunlechner R Hinterhuber H. Schizophrenie und Gewalttätigkeit Ergebnisse einer Gesamterhebung in einem österreichischen Bundesland. Der Nervenarzt. (2001) 72:859–66. doi: 10.1007/S001150170020, PMID: 11758093

[36] Müller JL Saimeh N Briken P Eucker S Hoffmann K Koller M. Standards for treatment in forensic committment according to § 63 and § 64 of the German criminal code: Interdisciplinary task force of the DGPPN. Nervenarzt. (2017) 88:1–29. doi: 10.1007/S00115-017-0382-3/TABLES/2, PMID: 28776213

[37] Fazel S Wolf A Palm C Lichtenstein P. Violent crime, suicide, and premature mortality in patients with schizophrenia and related disorders: a 38-year total population study in Sweden. Lancet Psychiatry. (2014) 1:44–54. doi: 10.1016/S2215-0366(14)70223-8, PMID: 25110636 PMC4124855

[38] Müller-Isberner R Born P Eusterschulte B Eucker S. Praxishandbuch Maßregelvollzug : Grundlagen, Konzepte und Praxis der Kriminaltherapie. Berlin, Medizinsiche Wissenschaftliche Verlagsgesellschaft (2017).

